# IMPLEMENTING HOME SAFETY TOOLKIT TO CAREGIVERS OF VETERANS WITH DEMENTIA

**DOI:** 10.1093/geroni/igz038.913

**Published:** 2019-11-08

**Authors:** Scott A Trudeau

**Affiliations:** American Occupational Therapy Association, Bethesda, Maryland, United States

## Abstract

The purpose of this project was to study the processes necessary to make a Home Safety Toolkit (HST) for Veterans with dementia accessible to veterans and their caregivers. This Type 3 Implementation–Effectiveness Hybrid Research Design, included diagnostic analyses of the current processes by which Veterans receive home safety items, and identification of modifications necessary in order to provide the HST to Veterans with dementia. Two Veterans Health Administration Networks, one in the Northeast and one in the Mid-Atlantic region, participated. A formative evaluation used semi-structured interviews with key staff informants and caregivers identified facilitators and barriers to successful acquisition and use of home safety items. Qualitative data analysis reveals key barriers of time and cost, selection of best items, and caregiver reluctance to change. There was resounding support from caregivers regarding the potential benefits of self-paced toolkit including education and home safety items to implement for their veteran.

